# Managing type 2 diabetes or prediabetes and binge eating disorder: a qualitative study of patients’ perceptions and lived experiences

**DOI:** 10.1186/s40337-022-00666-y

**Published:** 2022-10-12

**Authors:** Meg G. Salvia, Marilyn D. Ritholz, Katherine L.E. Craigen, Paula A. Quatromoni

**Affiliations:** 1 Department of Health Sciences, Boston University, 635 Commonwealth Avenue, 02215 Boston, MA USA; 2 Department of Nutrition, Harvard T.H. Chan School of Public Health, 677 Huntington Avenue, 02115 Boston, MA USA; 3 Joslin Diabetes Center, 1 Joslin Place, 02215 Boston, MA USA; 4 Department of Psychiatry, Harvard Medical School, 401 Park Drive, 02215 Boston, MA USA; 5 Walden Behavioral Care, 51 Sawyer Road, 02453 Waltham, MA USA

**Keywords:** Binge eating disorder, Type 2 diabetes, Prediabetes, Qualitative research, Eating disorder treatment

## Abstract

**Background:**

The overlap in prevalence between type 2 diabetes and binge eating disorder is substantial, with adverse physical and mental health consequences. Little is known about patients’ efforts at managing these two conditions simultaneously. The research objective was to explore patients’ experiences managing co-existing type 2 diabetes or prediabetes and binge eating disorder.

**Methods:**

This is a qualitative descriptive study using semi-structured interviews. Participants included 21 women with type 2 diabetes or prediabetes (90% non-Hispanic White; mean age 49 ± 14.8 years, mean BMI 43.8 ± 8.4; 48% with type 2 diabetes and mean HbA1c was 8.4%). Interviews were analyzed using thematic analysis and NVivo software.

**Results:**

Qualitative analysis revealed that participants reported binge episodes frequently started in childhood or adolescence and went undiagnosed for decades; notably, they recalled that diabetes diagnosis preceded the binge eating disorder diagnosis. They also described trying to lose weight throughout their lives and how feelings of deprivation, shame, and failure exacerbated binge eating. Participants further reported how binge eating made diabetes self-care and outcomes worse. Finally, participants observed that when binge eating disorder treatment and diabetes management were synergistically integrated, they experienced improvements in both binge eating and glycemic outcomes. This integration included reframing negative thoughts surrounding binge eating disorder and diabetes self-management and increasing their understanding of how the two disorders were inter-related.

**Conclusion:**

Findings highlight the importance of increasing healthcare providers’ awareness of and screening for binge eating disorder in the treatment of diabetes and inform specific integrated interventions that address both diagnoses.

**Plain English summary:**

From this study where we interviewed 21 women with binge eating disorder (BED) and type 2 diabetes/prediabetes, we learned how binge eating impacted diabetes management and how diabetes impacted BED. Most participants reported receiving the diabetes diagnosis before being diagnosed with BED despite the earlier onset of binge eating, pointing to the need for BED screening. Participants described trying to lose weight throughout their lives and reported feelings of failure and shame, which made binge eating worse. Binge eating made diabetes management harder, but when diabetes and BED treatment were aligned, participants experienced improvements in binge symptoms and diabetes outcomes.

## Background

The prevalence and public health challenges associated with type 2 diabetes (T2DM) and prediabetes are well-known [1]. Less well understood is the extent to which binge eating disorder (BED) occurs alongside T2DM and prediabetes and how its co-occurrence affects treatment recommendations and outcomes. T2DM is a chronic condition in which the body’s ability to use insulin decreases (insulin resistance), and over time, the ability of the pancreas’s beta cells to produce enough insulin to meet the demand declines, resulting in high blood glucose or hyperglycemia. BED is characterized by recurrent binge episodes in which an individual eats an objectively large amount of food in a discrete time with associated hallmark feelings of loss of control, distress, guilt, and shame but without compensatory purging behaviors [2]. Approximately 8% of U.S. adults have diagnosed T2DM [1], and BED is prevalent in about 2–3% of the general population [3].

Estimates of the prevalence of eating disorders among persons with T2DM vary. Studies suggest 7–20% of those with T2DM have binge eating behaviors [4–10] with 8% having a clinical diagnosis of BED [10], while in a population of people with BED, approximately one-third had T2DM [8]. Binge eating negatively impacts glycemic management of T2DM [11, 12], and it is unclear how individuals manage these two conditions or what specific challenges they face. There is mixed evidence regarding the directionality of the association between binge eating and T2DM. Persons with diabetes are at increased risk of disordered eating behaviors [8, 12]; and the prescribed or perceived rigidity of diet and weight loss recommendations for prevention and management of T2DM can exacerbate disordered eating among those with or predisposed to eating disorders [13–15]. Studies have also found an association between disordered eating and incident metabolic syndrome and T2DM [16], and youth with disordered eating behaviors had lower insulin sensitivity compared with those who exhibited normal eating patterns [17].

For individuals who experience both T2DM/prediabetes and BED, conflicting recommendations in medical and nutritional management present daily challenges. For example, diabetes prevention and management recommendations, such as those based in the National Diabetes Prevention Program [18], typically encourage weight-loss via energy deficits to support insulin sensitivity and delay disease progression [19]. However, BED treatment recommendations often encourage calorically adequate, balanced, and varied eating patterns with intentional therapeutic exposure to specific foods to reduce the hunger and dietary restraint that predispose to binge episodes. How patients and healthcare providers navigate these different treatment priorities requires better insight. The aim of this study was to qualitatively explore the experiences of women concurrently managing T2DM/prediabetes and BED to increase healthcare providers’ understanding of patients’ challenges and to inform the development of practical treatment strategies. We previously reported qualitative findings describing the experiences of women with these dual diagnoses in the primary healthcare setting [20]. Here, we report the individuals’ lived experiences with diabetes and BED onset, and their perceptions of self-management challenges.

## Methods

### Sample

Adult participants were recruited from an eating disorder treatment center in Massachusetts that offered intensive outpatient programming specifically for BED. The multidisciplinary team that provided BED treatment included a Registered Dietitian Nutritionist (RDN) trained in medical nutrition therapy which includes nutritional management of diabetes. Medical records from admissions between 2015 and 2019 were screened for co-occurring T2DM. Because binge eating has been associated with metabolic syndrome [21], we also recruited those with prediabetes (defined as “prediabetes,” “metabolic syndrome” or “impaired glucose tolerance” noted in the medical record). Additional inclusion criteria required completion of more than 2 weeks of the treatment program, discharge at least six months prior to study enrollment, and the ability to participate in an English-spoken interview. Exclusion criteria consisted of cognitive impairment or severe psychopathology limiting the ability to recall program participation or engage in the interview, or discontinuing the intensive outpatient program to admit to a higher level of care. Participants were invited by mailed letters.

Participants attended one hour-long study visit at the clinic. All participants were told of the research objectives, provided informed consent, and received monetary compensation ($50 gift card) for their participation. Study procedures were approved by the Boston University Institutional Review Board.

### Data collection

In-person interviews were conducted by MGS and PAQ, using a semi-structured interview guide outlining core research questions and follow-up prompts. The researcher conducting the interview did not have prior research or clinical relationships with participants. Interviews were audio-recorded and transcribed verbatim. Blinded weights were obtained at the study visit using the clinic’s calibrated scale. Biomedical data were obtained by self-report at the time of the interview and from medical records from outpatient providers. Participants gave consent separately to retrospectively request medical records from their outpatient providers. The questionnaire version of the Eating Disorder Examination (EDE-Q), designed to assess the range and severity of problematic eating behaviors and attitudes [22–24], was self-administered along with a brief survey to collect demographic data and diabetes management information.

### Data analysis

Interview transcripts were coded and analyzed by a multidisciplinary research team that was diverse in terms of disciplines, ages, and years and types of experiences. It included four women ranging in age from 38 to 74 years with training in nutrition, epidemiology, and psychology with up to 35 years of clinical and research expertise including behavioral, psychological, and medical nutrition therapy for eating disorders and diabetes. This team approach ensured investigator triangulation and supported the internal validity of the data [25]. The team met over the course of 12 months to analyze data according to the principles of thematic analysis [26]. Team members independently read transcripts and coded the data by marking and categorizing key words and phrases, using an iterative approach whereby codes were continuously revised and refined throughout the analysis. Importantly, through intensive discussion of codes including robust exploration of possible biases contributing to codes, the coding guide was revised more than ten times. This iterative analysis continued until data saturation for each theme occurred. After transcripts were coded and reviewed, one member of the research team (MGS) entered the marked transcripts into NVivo software [27] to organize and facilitate the grouping of codes into themes. An audit trail tracked the decision-making process and supported the dependability (reliability) of the data.

## Results

### Sample characteristics

**Fig. 1:**
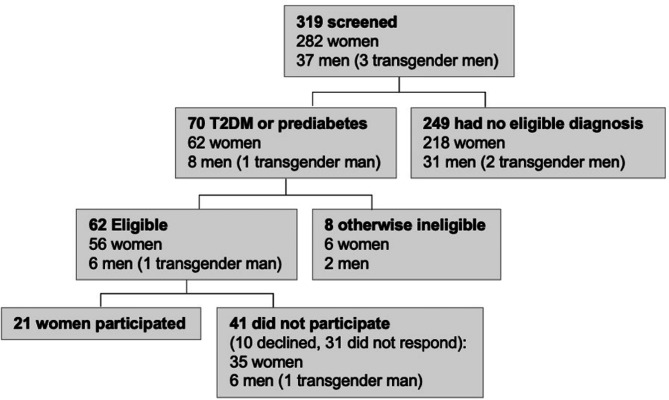
Screening and recruitment of BED patients

In our sample of 319 BED patients, 22% had either T2DM (n = 25) or prediabetes (n = 45). Of these, 8 did not meet further eligibility criteria, 10 declined, and 31 did not respond (see Fig. 1). Twenty-one participants were interviewed; of these, all were women. Participant characteristics are shown in Table 1.

**Table 1:** Participant characteristics (n = 21 women)

	Mean (range)	N (%)
Participant age, in years		
Mean (range)	49 (19–66)	
Participant race		
White		19 (90)
Black or African American		1 (5)
Multiple race identities indicated		1 (5)
BMI		
Mean (range)	43.8 (30.2–63.9)	
30.0-39.9		7 (33)
40.0-49.9		10 (48)
50.0-59.9		3 (14)
60.0-69.9		1 (5)
Education history		
Completed high school		1 (5)
Some college or technical school		6 (29)
College degree		11 (52)
Graduate degree		3 (14)
Employment status		
Employed, full-time		10 (48)
Employed, part-time		5 (24)
Disabled, employed part-time		1 (5)
Disabled, not able to work		4 (19)
Student		3 (14)
Insurance plan (at time of interview)		
Medicaid or Medicare		5 (24)
Group/private insurance through employer		13 (62)
Individual private insurance (self-purchased)		3 (14)
Treatment team composition (at time of interview)		
Primary care (physician or nurse practitioner)		21 (100)
Therapist		11 (52)
Dietitian		4 (19)
Endocrinologist		7 (33)
Diabetes educator		1 (5)
Psychiatrist		8 (38)

Diabetes characteristics and outcomes are presented in Table 2. Almost three quarters of participants reported binge eating onset in childhood or adolescence. Persistence of binge behaviors is noted with varying degrees of frequency in this population that received treatment for BED. EDE-Q data do not suggest high clinical concern of the eating disorder at the time of interview, though some individual scores were elevated, and weight and shape concern subscales trended the highest.

**Table 2:** Participants’ diabetes and binge eating outcomes (n = 21)

Characteristic	Mean (range)	N (%)	
Diabetes diagnosis			
Type 2 diabetes		10 (48)	
Current hemoglobin A1c (%)	8.4 (6.4–14.0)		
Diabetes duration (years)	12 (5–18)		
Insulin use (% yes)^*^		3 (15)	
Non-insulin T2DM medication use (% yes)^*^		9 (45)	
Metformin		8	
Sulfonylurea		2	
Thiazolidinedione		1	
DPP-4 inhibitor		1	
GLP-1 receptor agonist		1	
SGLT2 inhibitor		1	
Prediabetes		11 (52)	
Years since prediabetes diagnosis	5.7 (1–19)		
Number progressing to T2DM diagnosis		1 (9)	
Age/Life stage of binge onset (self-reported)			
Childhood		11 (52)	
Adolescence		4 (19)	
Young adulthood		4 (19)	
Middle adulthood		2 (10)	
Frequency of binge eating episodes (at time of interview)			
< 1x/month		5 (24)	
1-2x/month		2 (10)	
1x/week		4 (19)	
2-3x/week		3 (14)	
4-6x/week		2 (10)	
1x/day		3 (14)	
> 1x/day		2 (10)	
EDE-Q scores^**^			
Global Score	2.6 (0.6–5.3)		
Restraint	1.5 (0–3.6)		
Eating concern	2.5 (0–6)		
Shape concern	3.1 (0.3–6)		
Weight concern	3.5 (0.5–6)		

^*^One participant with T2DM declined to list medications; the sample size for the medication data points is 20. Some reported taking multiple non-insulin diabetes medications. Abbreviations include: sodium-glucose cotransporter-2 (SGLT2), dipeptidyl peptidase 4 (DPP-4), glucagon-like peptide-1 (GLP-1)

^**^EDE-Q global and subscale scores range from 0–6; higher number indicates more problematic eating behaviors and attitudes; a cut-off of 4 on the global score generally represents clinical significance [28]

### Qualitative themes

We identified four central themes in our qualitative analysis: (1) Early Onset and Delay in Diagnosis of Binge Eating, (2) Dieting Drives Binge Patterns; (3) Bidirectional Impact of DM and BED; and (4) Need for Synergy in Treatment Recommendations. Illustrative quotations are given with participant research number, T2DM or prediabetes status, and diabetes medication (insulin or class of oral medication) used or not used.

### Early onset and delay in diagnosis of binge eating

Most study participants reported that binge eating behaviors preceded the T2DM/prediabetes diagnosis, often by several decades. Many reported binge eating in childhood or adolescence:

I mean, I’ve binged since I was a kid. I’m not sure I had an official diagnosis that I was aware of, but I was aware that I binged.

– Participant #5, age 66, T2DM, no reported diabetes medication

This [binge eating] started about 8th grade, I was about 12, 13. Fourteen years old is when it got really bad… I was researching … and I found out about binge eating disorder, and I realized I fit a lot of the symptoms, but I didn’t really tell anyone about it because I was ashamed.

– Participant #19, age 22, prediabetes, no reported diabetes medication

Only two participants described BED developing later in life and cited mid-life changes and increased loneliness as factors in binge eating onset:

It wasn’t really until my 50’s, when my kids were gone out of the house, and I was on my own, and I didn’t have anybody there. I had a very busy lifestyle raising three kids. And that, I think, kept me from bingeing and giving in to food because they always filled up my life.

– Participant #12, age 66, T2DM, using insulin

Most participants also described delays in getting diagnosed with BED, spending much of those early decades without treatment. Many participants reported they were only diagnosed with BED when they chose to disclose behaviors to their medical team or therapists with the goal of seeking admission to a BED treatment program, which they’d often identified on their own from an internet search. In most cases, receiving the BED diagnosis was not as frightening to participants as the diabetes diagnosis was. In fact, the BED diagnosis was a source of relief and validation, opening up the possibility of treatment for what they now understood was an eating disorder.

### Dieting drives binge patterns

Participants frequently mentioned longstanding efforts at dieting or limiting intake of food (particularly sources of sugar and other carbohydrates) in their weight-loss efforts, which occurred prior to and concurrent with diabetes diagnoses. Participants described oscillating between windows of controlling intake with windows of binge episodes. Weight loss was also a priority articulated in health-care discussions with providers and family members. Some described feeling like their efforts at dieting or food restriction were related to increased binge eating. Participants also described early-life dieting as contributing to a sense of deprivation, followed by the guilt or shame associated with binge eating. These experiences contributed to repeating cycles of restrict-binge-restrict:

The weight definitely influenced the binge eating disorder because I would do everything, all my eating and all my exercise was to the goal of losing weight to look thin. … I would eat so many veggies and so many low-carb, low-fat, whatever… and later I would binge and feel bad about it, and those foods that I do like felt like demons.

– Participant #17, age 22, prediabetes, no reported diabetes medication

I thought about what led to my binge eating because that’s not a behavior I would ever have thought I would have. And I think, I really do think that the restriction is what made me start to binge.

– Participant #10, age 57, prediabetes, no reported diabetes medication.

### Bidirectional impact of T2DM and BED

#### Binge eating behaviors were responsible for development of diabetes

Many participants felt that binge eating directly contributed to their diabetes and prediabetes; they endorsed feelings of guilt and shame around the diabetes diagnosis associated with fears that they “did this to themselves” because of disordered eating behaviors.

Weight and shape influenced the course of my binge eating, which influenced the course of my prediabetes. I bet my binge eating created the prediabetes.

– Participant #16, age 63, prediabetes, no reported diabetes medication

I was not able to control my binge eating, that’s basically how I got diabetes… I think that type 2 diabetes also has some stigma attached to it that I’ve struggled with, like personal shame of ‘you brought this on yourself’ … So, there’s sort of some embarrassment and shame that goes along with that.

– Participant #18, age 63, T2DM, diabetes oral medications (metformin, dipeptidyl peptidase 4 (DPP-4) inhibitor, and sulfonylurea).

#### Heterogeneous impact of diabetes on binge eating

Participants with diabetes described the diagnosis experience as “scary” and prompted new realizations for the health implications of binge eating. Initially, some participants attempted to change eating behaviors to meet the demands and expectations of improved glycemic management, but they also described these changed eating behaviors as unsustainable:

The stakes were higher [with diabetes diagnosis], and when I failed it was more devastating. When I was first diagnosed [with diabetes] I was scared, and I would go through periods of being scared and controlling my eating really well and thereby controlling my blood sugars really well. And the problem was it didn’t last more than 2, 3, maybe 4 months. And then it regressed.

– Participant #13, age 59, T2DM, diabetes oral medication (metformin).

Others, however, reported immediate increases in binge episodes in response to the stress of the diabetes diagnosis. Compared to those with diabetes, participants with prediabetes expressed less concern about the way binge eating impacted their current glycemia or future T2DM risk.

Furthermore, some participants noted that, as occurred with weight-loss efforts, diabetes prevention/management recommendations were sometimes perceived as rigid, which could trigger black-and-white thinking and increase binge-eating episodes:

I did feel a little bit of the rules coming back [with diabetes diagnosis] where I hadn’t had food rules. And I think just knowing that I have those, and they make me nervous, and it just triggered a lot of the old patterns of thinking and behaviors.

– Participant #21, age 50, prediabetes progressed to T2DM, diabetes oral medication (metformin)

#### Binge eating made diabetes outcomes worse

Participants, especially those with T2DM, described binge episodes negatively impacting glycemic management by causing chronically elevated blood glucose levels that were difficult to bring down to goal ranges. Most participants described how difficult it was to interrupt binge cycles or reduce binge behaviors on their own even though they greatly feared the medical consequences of not improving their diabetes outcomes:

It disables me. The binge eating is the crux of it all. Like, if I could just stop that, managing diabetes would not be a problem. But because I have such an unhealthy attachment to food, I can’t stop. You know, I can’t stop because I’m gonna die, because I’m gonna lose my feet, because I’m gonna lose my sight… it still isn’t enough, and that’s pretty pathetic.

– Participant #11, age 56, T2DM, using insulin

It was very clear to me that those [BED] behaviors were making my diabetes and numbers worse. And it felt like this awful conundrum, that I need to treat the diabetes, but I can’t treat the diabetes until I deal with the eating, and the eating is making the diabetes worse. It felt like a diabolical combination. The two things were just intertwined and interlocked, and each was making the other worse. I don’t know that the diabetes made my binge eating worse, it was always there. But it was just a bad combination because I couldn’t just deal with it by taking a medication. I didn’t want to go on insulin. Very much didn’t want to go on insulin because, first of all, it would be acknowledging that my condition was getting worse, and I felt it would make my life more complicated.

– Participant #13, age 59, T2DM, diabetes oral medication (metformin)

I think that they both [bingeing and glycemia] kind of went hand in hand, definitely. The bingeing, the binge eating disorder – the, you know, restricting, bingeing, restricting, bingeing – definitely led to my blood sugar being [out of] whack.

– Participant #17, age 22, prediabetes, no reported diabetes medication.

#### Self-care and diabetes management decreased in response to binges

Most participants who self-monitored blood glucose described skipping glucose checks after bingeing as a means of avoiding information and feelings of shame and self-denigration. They reported not wanting to have elevated numbers provide visible evidence of the binges to themselves or to their diabetes treatment teams:

You don’t want to look at that [high glucose] number because you know it was your fault. You did it, and you could’ve avoided it… I would be afraid to look at the number because it would confirm that I’m a failure.

– Participant #18, age 63, T2DM, diabetes oral medications (metformin, DPP-4 inhibitor, and sulfonylurea).

This avoidance also occurred in the setting of sharing food records with the diabetes team:

To be honest, I wasn’t bringing [bingeing] up to my doctor, nor to the nutritionist. I was not talking about it… there would be many times when I did not have a good food log for her because I was refusing to write down how much I was eating, and it was fear. I just didn’t want to face it.

– Participant #16, age 63, prediabetes, no reported diabetes medication.

In terms of diabetes medications, participants endorsed adherence, reporting taking oral medications and insulin as prescribed. Notably, some described estimating an insulin dose in an effort to try to cover the binge episode despite not knowing the carbohydrate content of the binge:

If I’m going to be on a binge, I’m going to be way above 350. So, I feel like I’m pretty safe at a 20 [unit] dose. Which is not what my – my endocrinologist would just flip out if she heard me say that because that’s not at all a responsible, healthy way to maintain your diabetes. And I do realize that, but it’s better than doing nothing.

– Participant #11, age 56, T2DM, using insulin.

#### Binge eating treatment improved diabetes self-care

Many participants reported that BED treatment increased their awareness of how BED and diabetes were connected. They reported learning how meal timing, nutritional content of foods, and binge patterns impacted their blood glucose levels. Some reported seeing improvements in diabetes control, such as fewer glycemic excursions and more consistent T2DM self-care, as binge behaviors decreased:

Managing the binge eating disorder helped me manage the prediabetes, which had taken more of the backburner… I really have been making a lot of progress. I binge so much less than before. I restrict so much less than before.

– Participant #17, age 22, prediabetes, no reported diabetes medication

Once I managed my binge eating, my numbers were much, much better, so doing this [treatment] in turn helped my diabetes… I don’t remember the numbers exactly, but I knew that I wasn’t seeing them in the 300 or 400s. That was good.

– Participant #11, age 56, T2DM, using insulin

### Need for synergy in treatment recommendations

Participants described experiencing conflict when they attempted to integrate diabetes and BED treatment recommendations which often differed. This contributed to frustration and confusion:

I was always going back and forth with, you know, working on trying to heal the eating disorder but then also freaking out: ‘but I have diabetes, I need to get serious about this, I need to buckle down, like, I’m doing damage to myself.’

– Participant #1, age 40, T2DM, diabetes oral medications (metformin, thiazolidinedione)

For example, some participants described a lack of cohesion when incorporating foods that were typically viewed as “off-limits” from the diabetes-management lens in the context of BED treatment. Specifically, the inclusion of carbohydrate-based foods into the meal plan designed to normalize eating patterns concerned some individuals and challenged treatment buy-in. Participants also experienced conflict when diabetes recommendations focused on caloric restriction for weight loss while BED treatment prioritized reducing or extinguishing bingeing, taking a weight-neutral approach. Some described these circumstances as stressful and this contributed to a decreased sense of well-being, particularly regarding diabetes self-care and psychosocial stress:

I remember one person in my group having a big bowl of ice cream and being like, what the…? How are we supposed to lose weight if they’re encouraging us to eat dessert? And then learning through getting to know her, that [ice cream] was one of her trigger foods, so she maybe at home used to eat a whole gallon, and now she’s having a big bowl, but it’s still less. And that everyone had a different kind of plan, and it was much more about the psychology of it than the calories.

– Participant #6, age 44, prediabetes, no reported diabetes medication

I just have this ingrained in my head that I’m not supposed to have dessert so that was really challenging for me. Kind of like very dichotomous... it’s like if I’m eating well, there’s no dessert; if I’m eating not well, there is dessert.

– Participant #10, age 57, prediabetes, no reported diabetes medication

In contrast, some participants described a process of treatment synergy occurring when providers explicitly accounted for both BED and diabetes diagnoses in treatment discussions and recommendations, resulting in self-reported improvement in glycemia. They described how first and foremost providers needed to be aware of both diagnoses and that when BED was kept secret and not openly disclosed, medical providers did not inquire about symptoms or incorporate BED into the treatment plan:

I think part of my denial before [BED] treatment was not getting the connection between my eating and my blood sugar levels.

– Participant #15, age 32, prediabetes, no reported diabetes medication

Similarly, participants noted how some BED providers had limited knowledge of diabetes care, compromising their ability to integrate diabetes-management goals.

Finally, participants stated that the integrated discussion of both diagnoses helped address the negative thoughts, self-judgment and self-blame that contributed to both binge eating and poor diabetes management:

I think they [BED and T2DM recommendations] were in sync about forgiving yourself, being kinder to yourself, giving you permission to fail and rebound… Because I do believe it’s the emotional beating yourself up that starts that vicious, vicious cycle of binge eating and feeling like a failure … I’m more consistent with [blood glucose] monitoring because, again, I try to tell myself it’s really just a number. It’s like a road map… rather than have it be a judgment. And it helps me to go in a different direction.

– Participant #18, age 63, T2DM, diabetes oral medication (metformin, DPP-4 inhibitor, and sulfonylurea)

## Discussion

In this qualitative study, we explored adult women’s experiences of living with and managing T2DM/prediabetes and BED. We found almost all participants reported that binge eating preceded their diagnosis of diabetes. Participants also voiced beliefs that their longstanding efforts at weight loss contributed to binge patterns, and that bingeing subsequently led to developing diabetes. They further described how binge eating made diabetes self-care more difficult. While they universally took diabetes medication as prescribed, many reported avoiding checking blood glucose levels to conceal the visibility of binge episodes for themselves and from their diabetes treatment team. Participants described the need for BED treatment and diabetes management to be synergistically integrated, and when this occurred, participants reported improvements in both binge eating and glycemic outcomes. Helpful treatment strategies included providing specific education to increase understanding of how the two conditions were connected, reframing negative thoughts surrounding BED and diabetes self-management while building appropriate behavioral and coping skills. To achieve this, screening for BED by medical providers and referral to multidisciplinary BED treatment professionals appear essential for those with diabetes/prediabetes, but perhaps more broadly for individuals with longstanding patterns of dieting or weight cycling. Patterns that participants self-identified in this study, such as gaps in blood glucose logs or food records, may indicate attempts to hide binge behaviors from themselves and from providers.

**Fig. 2:**
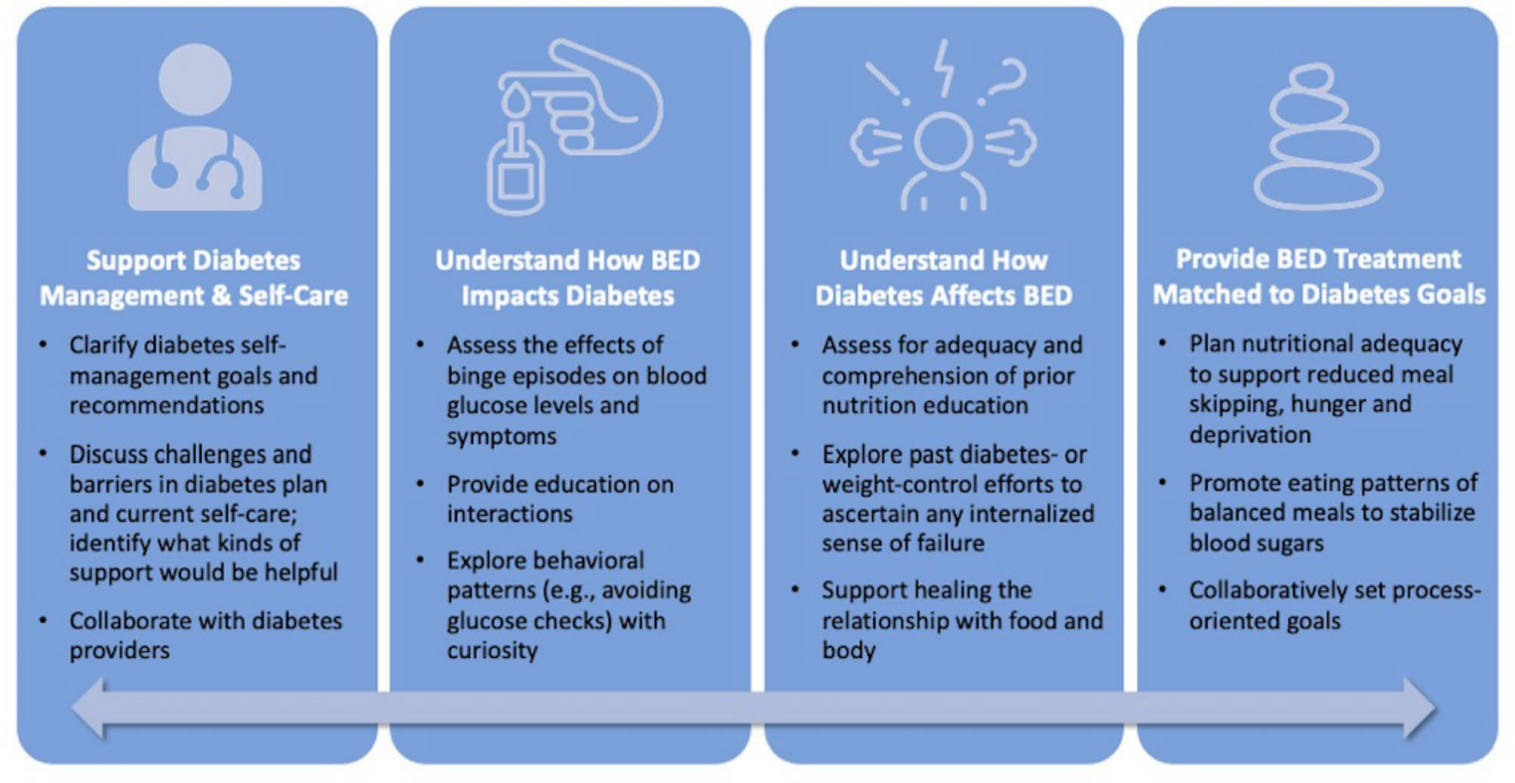
Strategies for synthesizing BED and T2DM treatment recommendations

Informed by our qualitative findings, strategies for synthesizing BED and T2DM recommendations are summarized in Fig. 2. Becoming familiar with the diabetes management goals outlined by the American Diabetes Association (ADA), including promoting and endorsing healthful eating patterns, improving overall health and well-being, and preventing or delaying diabetes complications, can equip eating disorder clinicians in supporting clients with both diagnoses. ADA recommendations emphasize patient-centered, individualized, and collaborative care to address individual needs [18, 19, 29]. BED treatment that reduces the frequency and severity of binge eating can additionally support improved glucose management. Medications for BED (lisdexamfetamine) and T2DM (GLP-1 agonists and SGLT-2 inhibitors) may be useful for supporting treatment goals for both conditions.

As in other studies [8, 15], we found that binge eating often preceded the diabetes diagnosis. However, most of our participants reported that the BED diagnosis did not occur until after they were first diagnosed with diabetes. One contributor to the delay in BED diagnosis may be the lack of familiarity with and assessment for BED among healthcare providers [30, 31]. Our finding underscores the importance of primary care and diabetes professionals strategically screening for BED as early as possible. Screening tools such as the BEDS-7 [32] can help identify those at greatest concern. Understandably, widespread screening may not be practical in all clinical settings. In the setting of T2DM, as has been observed in type 1 diabetes [33], it may be helpful for primary care or diabetes specialists and educators to, at a minimum, ask targeted questions when patterns of avoiding blood glucose checks are observed. For example, one might engage discussion by asking, “Can you tell me more about your eating habits on the days when you find yourself not doing glucose checks?” or “Do your eating patterns change when you eat alone compared to eating with others?” Studies have reported that individuals who experienced binge episodes were significantly younger at age of T2DM diagnosis compared to those without binge eating episodes [34]. Importantly, younger age of T2DM diagnosis increases the risk for adverse cardiovascular and mortality outcomes [35]. Collectively, these findings highlight the critical need for routine screening and early intervention for people with BED and T2DM [4, 36–38].

BED has been demonstrated to be a risk factor for the development of T2DM [39, 40]. Previous studies found binge eating high-fat and high-calorie foods contributed to decreased insulin sensitivity [41], higher fasting blood glucose levels, and increased insulin resistance [34]. Previous research in a population of women with T2DM [42] suggested the temporal order of BED and diabetes is that binge eating preceded both dieting onset and T2DM diagnosis. Our participants’ timelines followed this general pattern except they tended to report dieting preceded and may have precipitated the onset of binge eating. This difference in findings should be further explored to better understand the temporal order of bingeing, dieting and diabetes, and implications for effective treatment.

Participants also perceived BED as interfering with their diabetes management by contributing to decreased self-care behaviors. Avoidance of post-binge glucose monitoring was rooted in self-blame and shame. These feelings are salient contributors to patients’ inability or reluctance to self-disclose binge eating behavior and/or to ask providers for help. When healthcare professionals consider these seemingly non-adherent behaviors and approach them with curiosity and an awareness of the underlying shame and self-blame, they may foster useful conversations that invite patients to open up about their struggles. In another analysis from this same study sample, we previously reported that primary care providers who individualized diabetes education and facilitated non-judgmental, empathic conversations that showed an understanding of the difficulties managing BED and T2DM were perceived by patients as helpful [20]. Importantly, participants in our study reported adhering to prescribed oral medications and insulin doses even when experiencing binges. Thus, unlike women with type 1 diabetes and eating disorders who use insulin omission as a calorie-purging technique [43], our participants did not omit or manipulate medication for weight-loss purposes, though only three participants in our study used insulin.

After treatment for BED, most women reported reduced or extinguished binge eating behaviors in the short-term. This pattern was sustained post-treatment for some, but other participants continued to struggle with binge eating recurrence particularly around life changes and significant stressors. This suggests that for many, BED is a more chronic condition and that cycles of binge eating are a concern deserving ongoing evaluation in primary care settings. Because some women reported binge eating onset or relapse around significant mid-life changes, screening for BED can have important clinical relevance when women face events such as divorce, death of a spouse, experiencing an “empty nest,” or retirement.

Participants also voiced concerns about the conflict they experienced between BED and diabetes treatment recommendations. Although nutritional recommendations for T2DM have moved from strict prescriptions for carbohydrates, protein and fat to more individualized recommendations based on the assessment of patients’ eating patterns and personal preferences including culture, tradition, religion, access, health beliefs and personal goals [29, 44], weight loss is still a major focus of treatment for T2DM. Treatment of patients with BED and diabetes may require not only more flexible eating patterns and advice, but also an understanding that the diabetes nutrition plan should accommodate the eating disorder treatment plan [45] before weight loss is considered and evaluated for its therapeutic relevance [4]. BED eating plans that incorporate a proper balance of high-quality carbohydrate from a variety of food sources can be an educational and healing tool to support consistent, nourishing dietary adequacy and steady glycemic control. Further, introducing “challenge” or “trigger” foods (i.e., foods frequently included in binge episodes that elicit anxiety in other settings, which are often ultra-processed foods and/or high in refined carbohydrates), when exposed appropriately in the context of eating disorder treatment, offers an opportunity for individuals and their treatment team to learn about how their body responds to the incorporation of this food into their eating pattern. This curiosity allows the team to tailor diabetes recommendations with this insight and diminishes the power of the trigger food that is otherwise “off limits” but charged with temptation and dread. As with recommendations for eating disorders and T1DM [43], relaxing the glucose targets for diabetes treatment may allow patients with T2DM to engage more effectively in eating disorder treatment [4].

The finding that participants in our study reported improvements in glycemic control and reduced binge episodes with BED treatment is a noteworthy outcome, showing dual benefits of eating disorder treatment on both diagnoses. This observation warrants further study with larger and more diverse samples of persons diagnosed with BED and T2DM/prediabetes. More needs to be understood about how and why some participants sustain these improvements while others do not, as well as how BED treatment may influence the course of prediabetes and type 2 diabetes.

There is agreement among diabetes and eating disorder professional organizations that a multidisciplinary treatment team is the optimal approach for BED and diabetes [4, 44]. Ideally, the team includes a primary care medical provider and/or endocrinologist, mental health provider or psychologist, dietitian, diabetes educator, and sometimes a psychiatrist. This collaborative multi-disciplinary approach allows for an integration of diabetes and BED treatments, so patients experience knowledgeable and unified care. Of note, participants voiced that adequate expertise in and consideration for both BED and diabetes was helpful, when it existed. Some, but not all, participants reported having an integrated experience and described the absence of conflict in their treatment recommendations; this allowed them to better understand how BED and diabetes were connected.

There are limitations to this study that deserve consideration. The sample consisting of mostly non-Hispanic White well-educated women who were treated at a specialized eating disorder clinic limits the generalizability of the findings. The prevalence of BED among Black women and White men is like that among White women [46] and the prevalence of T2DM in minorities is far greater than in non-Hispanic Whites [1]. Thus, research on diabetes and BED needs to include more diverse populations. This presents real challenges to researchers since men and racial and ethnic minorities are less likely to seek treatment for BED [47]. The public health magnitude of this situation again endorses consideration of BED screening. Because we studied a treated population, we do not know about the experiences of adults who live with BED and T2DM who do not seek treatment. This caveat emphasizes the important role that primary care providers can play in screening for BED and directing patients to eating disorder treatment. Otherwise, the burden of awareness of appropriate, integrated treatment messages falls too heavily to the primary care team without the added assistance from eating disorder professionals. Similarly, because all participants in our study had a BMI of 30 or higher, we cannot discern whether some of the experiences and perceptions are related to weight, rather than the diabetes/prediabetes diagnoses in isolation. This is an area deserving targeted investigation. Finally, research also needs to investigate healthcare providers’ perspectives to better inform treatment strategies for co-occurring BED and T2DM/prediabetes. In addition, self-reported improvements in health outcomes were documented in this study, and larger quantitative studies are needed to confirm these findings.

## Conclusion

In summary, participants with BED and diabetes described struggling with efforts at weight loss for many years and how they perceived this struggle as contributing to both their binge eating and the development of diabetes. They also voiced concerns about how binge eating made diabetes management more difficult and how conflicting messages about the management of BED and diabetes were frustrating. Importantly, participants benefitted from treatment approaches that explained the connection between the two disorders and provided evidence of how improving one disorder contributed to improvement of the other. Our findings inform clinical suggestions that can empower healthcare providers to approach the treatment of BED and T2DM/prediabetes by adopting screening, referrals, and effective patient communication and education strategies.

## Data Availability

The dataset used and analyzed during the current study are not publicly available due to the sensitivity of the data (i.e., transcribed interviews from human subjects) and are available from the corresponding author upon reasonable request.

## List of abbreviations

T2DM: type 2 diabetes.
BED: binge eating disorder.
EDE-Q: Eating Disorder Examination questionnaire.
